# The value of ultrasound grayscale ratio in the diagnosis of papillary thyroid microcarcinomas and benign micronodules in patients with Hashimoto’s thyroiditis: A two-center controlled study

**DOI:** 10.3389/fendo.2022.949847

**Published:** 2022-08-12

**Authors:** Na Feng, Peiying Wei, Xiangkai Kong, Jingjing Xu, Jincao Yao, Fang Cheng, Di Ou, Liping Wang, Dong Xu, Zhijiang Han

**Affiliations:** ^1^ Department of Ultrasound, The Cancer Hospital of the University of Chinese Academy of Sciences (Zhejiang Cancer Hospital), Hangzhou, China; ^2^ Institute of Basic Medicine and Cancer (IBMC), Chinese Academy of Sciences, Hangzhou, China; ^3^ Department of Radiology, Affiliated Hangzhou First People’s Hospital, Zhejiang University School of Medicine, Hangzhou, China; ^4^ Department of Pathology, The Cancer Hospital of the University of Chinese Academy of Sciences (Zhejiang Cancer Hospital), Hangzhou, China; ^5^ Research Center for Cancer Intelligent Diagnosis and Molecular Technology, The Cancer Hospital of the University of Chinese Academy of Sciences (Zhejiang Cancer Hospital), Hangzhou, China; ^6^ Key Laboratory of Head & Neck Cancer Translational Research of Zhejiang Province, The Cancer Hospital of the University of Chinese Academy of Sciences (Zhejiang Cancer Hospital), Hangzhou, China

**Keywords:** ultrasound grayscale ratio, papillary thyroid microcarcinomas, nodular goiters, diagnosis, echogenicity, Hashimoto’s thyroiditis

## Abstract

**Objective:**

The value of ultrasound grayscale ratio (UGSR) in the diagnosis of papillary thyroid microcarcinomas (PTMCs) and benign micronodules (BMNs) has been recognized by some authors, but studies have not examined these aspects in patients with Hashimoto’s thyroiditis (HT). This retrospective study investigated the value of UGSR in the diagnosis of PTMCs and BMNs in patients with HT using data from two medical centers.

**Methods:**

Ultrasound images of 428 PTMCs in 368 patients with HT and 225 BMNs in 181 patients with HT in center A were retrospectively analyzed and compared to the ultrasound images of 412 PTMCs in 324 patients with HT and 315 BMNs in 229 patients with HT in medical center B. All of the cases were surgically confirmed. The UGSR was calculated as the ratio of the grayscale value of lesions to the surrounding normal thyroid tissues. The optimal UGSR thresholds for the PTMCs and BMNs in patients with HT from the two medical centers were determined using a receiver operating characteristic (ROC) curve. Furthermore, other statistics, including the area under the curve (AUC), the optimal UGSR threshold, sensitivity, specificity, positive predictive value (PPV), negative predictive value (NPV), and diagnostic accuracy of the two medical centers, were pair analyzed in this study.

**Results:**

The UGSR of PTMCs and BMNs in patients with HT from medical center A were 0.513 (0.442, 0.592) and 0.857 (0.677, 0.977) (*Z* = −15.564, *p* = 0), and those from medical center B were 0.514 (0.431, 0.625) and 0.917 (0.705, 1.131) (*Z* = −15.564, *p* = 0). For both medical centers A and B, the AUC, optimal UGSR threshold, sensitivity, specificity, PPV, NPV, and diagnostic accuracy of the UGSR in differentiating between PTMCs and BMNs in patients with HT were 0.870 and 0.889, 0.68 and 0.70, 0.921 and 0.898, 0.747 and 0.759, 0.874 and 0.829, 0.832 and 0.848, and 0.861 and 0.836, respectively. There were no significant differences in the UGSR for the PTMCs between patients from the two medical centers (*Z* = −0.815, *p* = 0.415), while there was a significant difference in the UGSR of the BMNs between patients from the two medical centers (*Z* = −3.637, *p* = 0).

**Conclusion:**

In the context of HT, UGSR still has high sensitivity, accuracy, and stability in differentiating between PTMCs and BMNs, making it a complementary differentiator of thyroid imaging reporting and data systems. However, due to its low specificity, a comprehensive analysis of other ultrasound signs is required.

## Introduction

Hashimoto’s thyroiditis (HT), also known as chronic lymphocytic or autoimmune thyroiditis, is the most common inflammatory thyroid disease. Its pathological basis is as follows: The thyroid is infiltrated by lymphocytes and plasma cells, and a lymphoid follicle with germinal centers appears, resulting in the gland undergoing parenchymal atrophy and fibrosing (1, 2). Ultrasound echo intensity reflects the internal pathological basis of HT. For example, lymphocyte and plasma cell infiltration decreases the echo intensity, and fibrosis causes heterogeneous echo intensity. Therefore, ultrasound has become the most important imaging method for evaluating and monitoring HT. Papillary thyroid carcinoma is the most common pathological subtype of thyroid carcinoma, usually coexisting with HT. HT is even considered an independent risk factor for papillary carcinoma (3, 4). Hypoechoic imaging is an important component in the diagnosis of papillary thyroid carcinoma, especially for papillary thyroid microcarcinoma (PTMC). According to the traditional ultrasound echo intensity grading scale, most authors (5–8) believe that ultrasound echo intensity has almost the same importance in differentiating between benign and malignant thyroid nodules regardless of the existence of HT. The proportions of hypoechoic imaging in malignant nodules in patients both with and without HT are 63.6%–78.5% (5, 6) vs. 65.9%–87.5% (5, 9), respectively. However, for benign and malignant nodules in patients with HT, due to lymphocytes’ and plasma cells’ different infiltration degrees, the echo intensity of the nodules and the surrounding normal thyroid tissue may vary. The results of echo intensity classification may not change as observers do not notice these minor differences with the naked eye. Therefore, the quantification of ultrasound echo intensity can identify these minor differences, thus providing a more objective basis for the classification of thyroid nodules.

Studies on the quantification of ultrasound echo intensity in terms of thyroid nodules include the directly quantified gray-scale histogram (10) and the indirectly quantified ultrasound grayscale ratio (UGSR), the latter of which is well recognized by the majority of scholars (11–16). Compared to the parameters of directly quantified gray-scale histograms, UGSR can effectively eliminate the unstable factors in gray-scale values that are caused by different ultrasound systems, operators, gains, and dynamics settings, and it uses a relatively stable ratio to indirectly quantify the echo intensity of nodules. In our previous studies (13–16), there are only six articles discussing UGSR (11–16), all of which suggest that the rates at which UGSR is used for malignant nodules are lower than those for solid benign nodules (11–15) but higher than those for cystic benign nodules (16). The diagnostic efficiency of UGSR is significantly higher than the traditional echo intensity grading scale. However, the current six articles have common deficiencies in that all of the samples comprised patients that did not have HT. Therefore, there is no relevant study on whether UGSR is effective in differentiating between benign and malignant thyroid nodules in patients with HT.

Through pair-analyzing UGSR and its diagnostic accuracy in two medical centers, this study’s objective is to evaluate the value and reproductivity of UGSR in differentiating between PTMCs and benign micronodules (BMNs) in patients with HT to provide a potential reference for improving thyroid imaging reporting and data systems (TI-RADS).

## Materials and methods

### Participants

**Figure 1:**
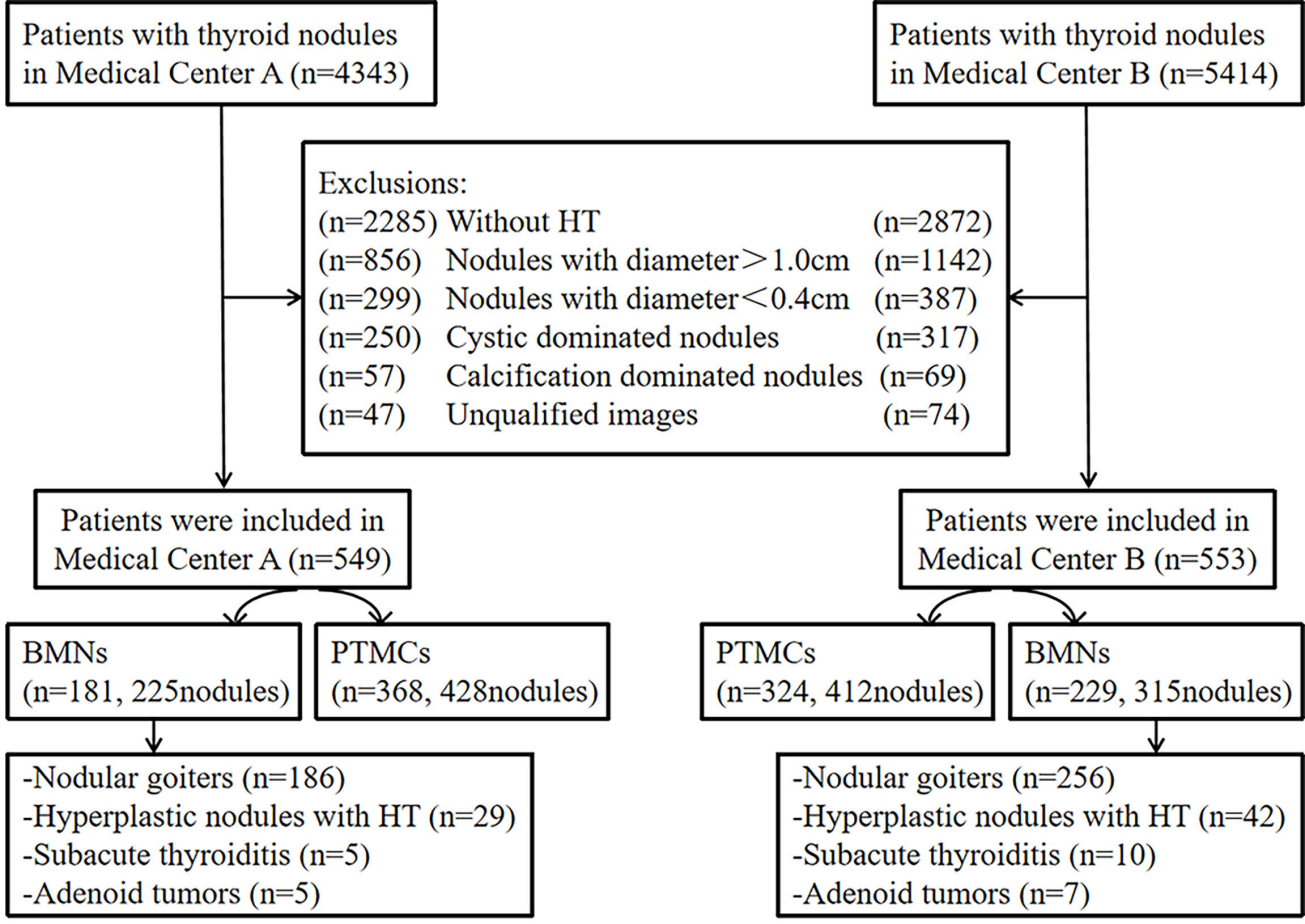
Flow chart of the study participants.

The study was performed in accordance with the ethical guidelines of the Helsinki Declaration. It was approved by the Ethics Committee of Affiliated Hangzhou First People’s Hospital (IRB-2019-200) and the Ethics Committee of Zhejiang Cancer Hospital (IRB-2020-287). Due to the retrospective nature of the study and the use of anonymized patient data, written informed consent for participation was waived. We identified a total of 4,343 consecutive patients with thyroid nodules who were treated in medical center A (the Affiliated Hangzhou First People’s Hospital, Zhejiang University School of Medicine) and 5,414 consecutive patients with thyroid nodules who were treated in medical center B (the Cancer Hospital of the University of Chinese Academy of Sciences, Zhejiang Cancer Hospital) from January 2018 to February 2022. The inclusion criteria were as follows: (1) patients had to have thyroid nodules complicated with HT, confirmed by both surgery and pathology; (2) all cases had to include a preoperative ultrasound examination. The following types of nodules were excluded: (1) nodules with a maximum diameter of >1.0 or<0.4 cm; (2) cystic-dominated nodules in which the cystic component was greater than 50% of the nodule volume (9); (3) calcification-dominated nodules in which the nodules could not be measured due to obvious calcification (12, 13); (4) nodules without thyroid peroxidase antibodies (TPO-Ab) and antithyroglobulin antibodies (TG-Ab) upon examination; (5) unqualified images, including a heterogeneous echo of the thyroid or nodules due to operators’ technical factors, diffuse hypoechoic nodules, and insufficient residual thyroid for grayscale values measurement, and more. This resulted in 1,102 patients with 1,380 thyroid nodules who met the inclusion criteria being included in the study. **Figure 1** shows the characteristics of the study participants in a flow chart.

### Ultrasonic examination

The following six models of ultrasonic diagnostic scanners were used in medical center A: MyLab 70 XVG (Genova, Italy), Esaote MyLab Classic C (Genova, Italy), Esaote MyLab 90 (Genova, Italy), Mindray (Shenzhen, China), Hitachi (Tokyo, Japan), and Philips EPIQ 5 (Washington, USA). The following four models of ultrasonic diagnostic scanners were used in medical center B: Toshiba Aplio 400 (Tochigi, Japan), GE Logiq E9 (Wauwatosa, USA), Siemens Acuson Sequoia (Auburn Hills, USA), and SonoScape S60 VO (Shenzhen, China). In this study, 5–10 MHz broadband linear array probes were used, with the central frequency being 7.5 MHz.

All patients were in the supine position, with their necks fully extended to the far back so that the anterior cervical area was fully exposed. Ultrasonic scanning of lesions was performed in transverse sections, longitudinal sections, and other sections. The number of nodules and their size, shape, boundary, surrounding acoustic halo, internal echo, calcification, and internal and peripheral blood supply as well as the bilateral neck lymph nodes were recorded.

### TPO-Ab and TG-Ab tests

Thyroid function tests were performed in all cases before obtaining pathological tissues, and the TPO-Ab and TG-Ab values were recorded. Chemiluminescence immunoassays through the Siemens ADVIA Centaur XP System (Siemens Medical Diagnostics Inc., USA) were used in both medical centers.

### Pathology

Tissue specimens were made into 5 μm sections with 10% neutral buffered formalin fixative and hematoxylin–eosin stains in both medical centers. Finally, these sections were examined under a light microscope, and all thyroid nodules were pathologically confirmed.

### Image analysis

**Figure 2:**
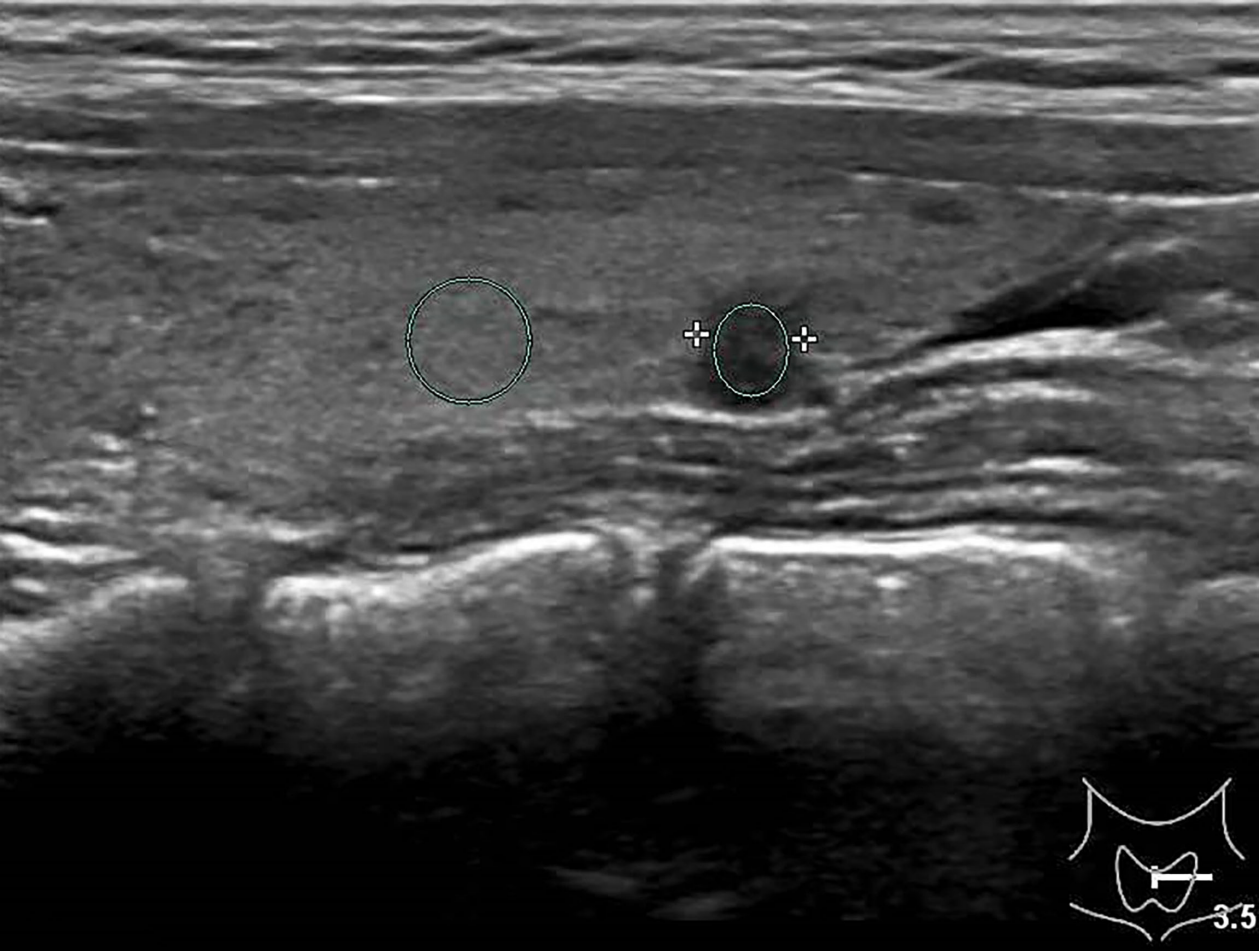
Female patient, 32 years old, 8-year case history of a thyroid nodule, pathology confirmed as PTMC with HT. TPO-Ab = 90.2 kU/L, TG-Ab>500 kU/L, UGSR = 43.18/95.61 = 0.4516 (medical center A).

**Figure 3:**
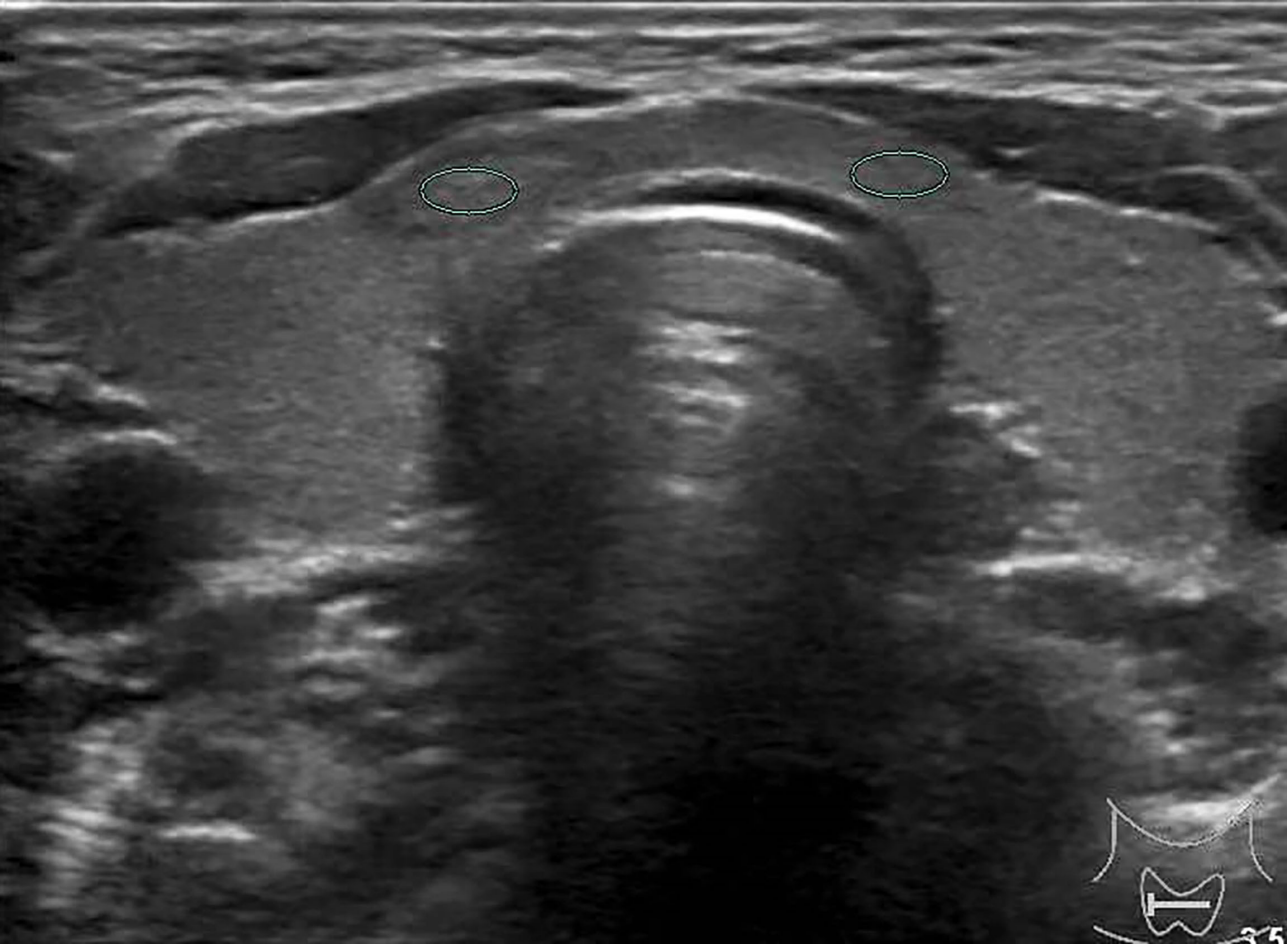
Female patient, 37 years old, 1-year case history of a thyroid nodule, pathology confirmed as nodular goiter with HT. TG-Ab = 1005.2 kU/L, TPO-Ab = 109.5kU/L, UGSR = 84.76/77.1 = 1.09945 (medical center B).

**Figure 4:**
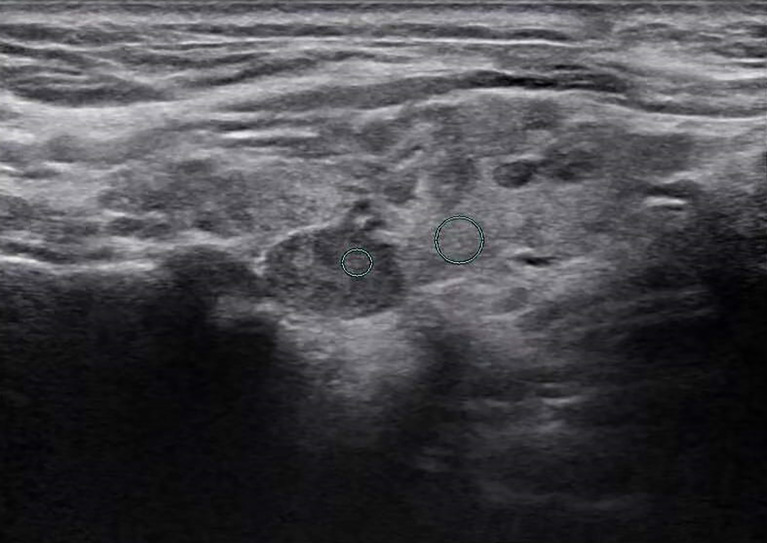
Female patient, 60 years old, 5-year case history of a thyroid nodule, pathology confirmed as adenomatous goiter with HT. TPO-Ab = 28.3 kU/L, TG-Ab>500 kU/L, UGSR = 78.73/105.3 = 0.7477 (medical center A).

**Figure 5:**
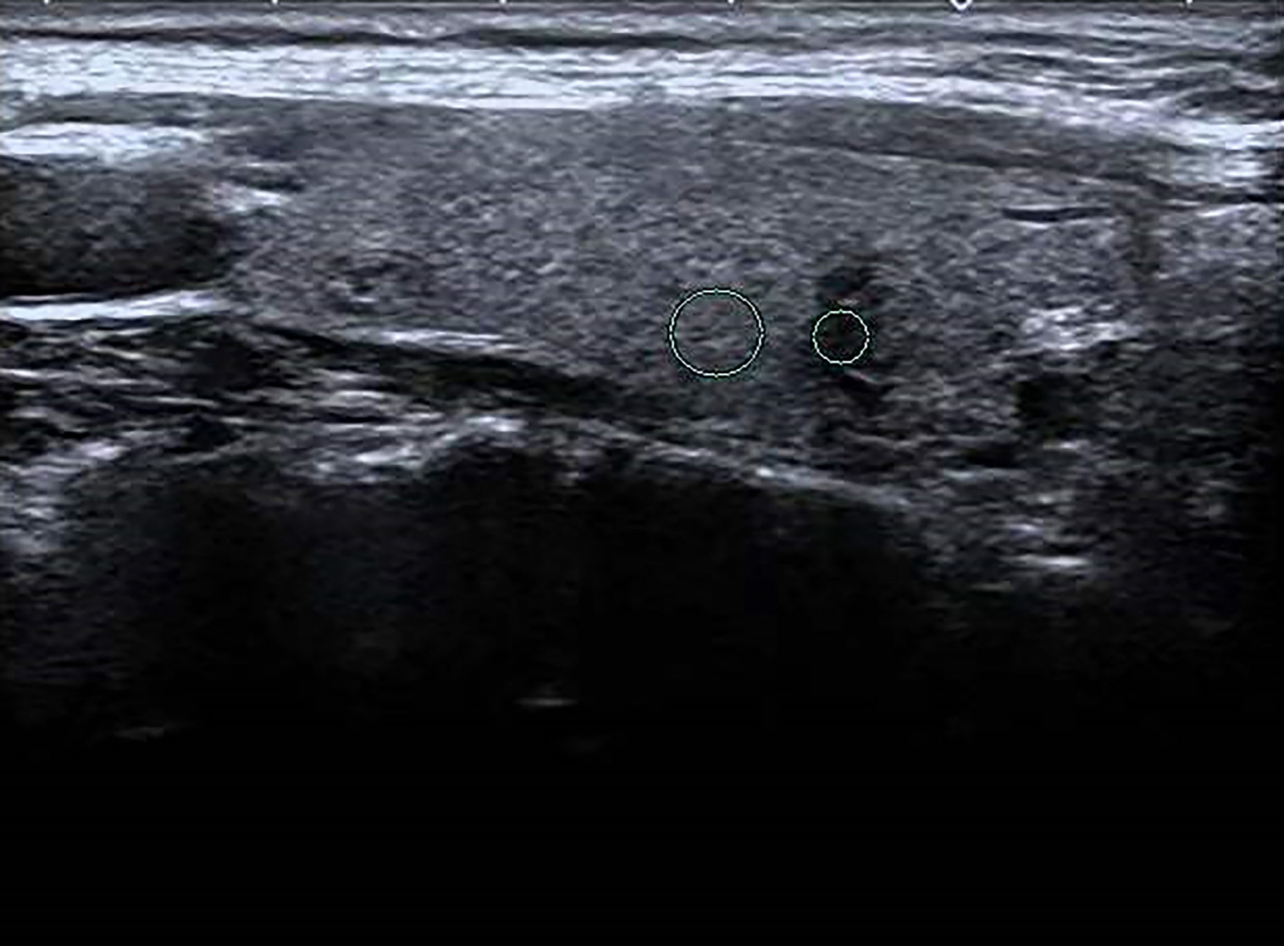
Female patient, 45 years old, 1-week case history of a thyroid nodule, pathology confirmed as a hyperplastic nodule with HT. TG-Ab = 115 kU/L, TPO-Ab< 28 kU/L, UGSR = 22.96/73.95 = 0.3105 (medical center B).

**Figure 6:**
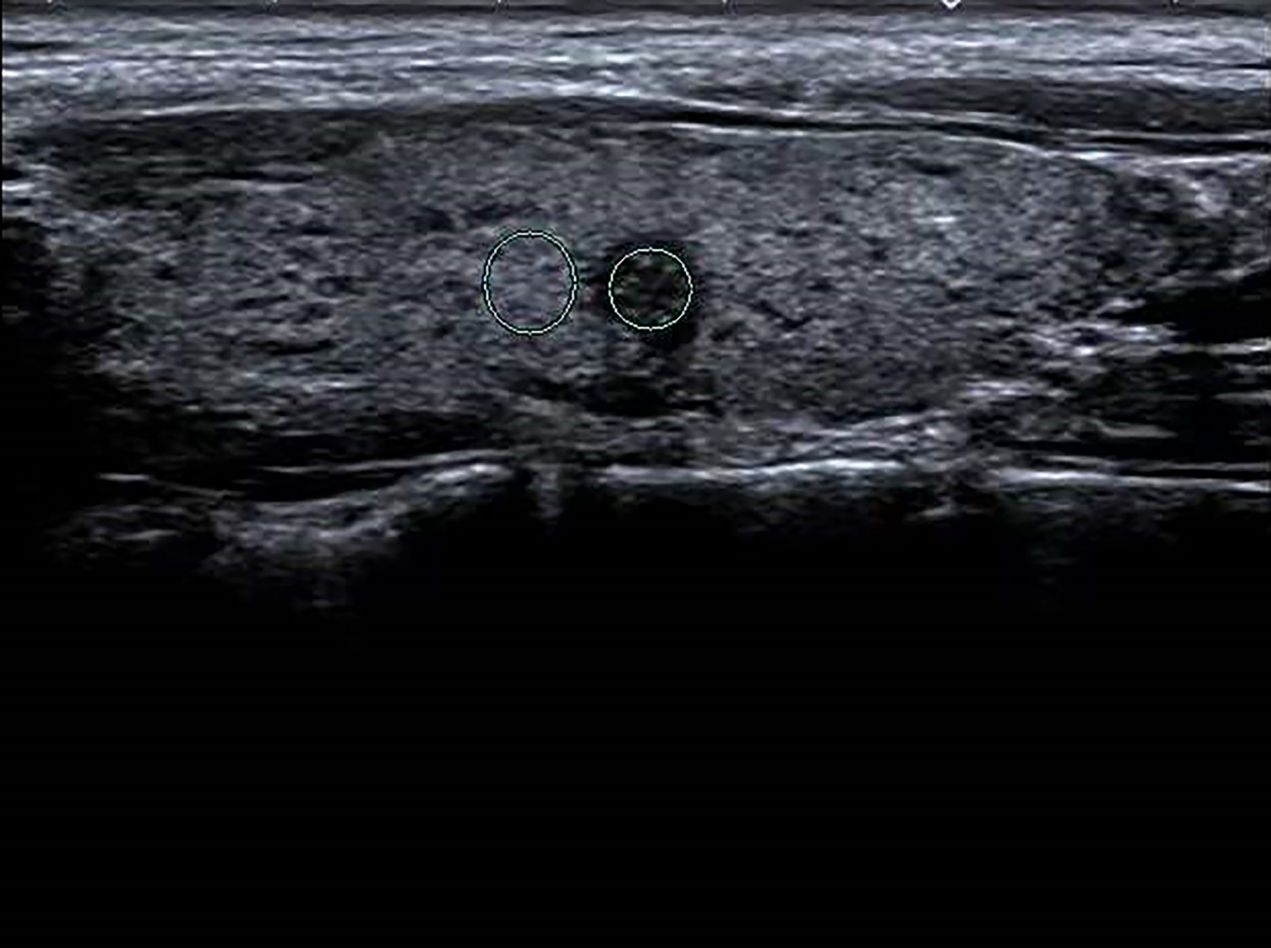
Female patient, 33 years old, half-month case history of a thyroid nodule, pathology confirmed as PTMC with HT. TG-Ab = 278 kU/L, TPO-Ab = 600 kU/L, UGSR = 30.04/85.33 = 0.352 (medical center B).

A radiologist with more than 10 years of experience in both medical centers, who was unaware of the pathological results, independently analyzed the selected cases of picture archiving and communication systems in order to determine the region of interest (ROI) location and the size of the thyroid nodules and their surrounding tissue. This was completed using gray-scale histogram software from RADinfo Systems (Zhejiang Rad Information Technology Co. Ltd., Hangzhou, China). The grayscale value of the nodules and their surrounding tissues were measured in ultrasound transverse or longitudinal section scanning. The ROI was as large as possible (**Figures 2**, **3**) when the echo intensity of the measured nodule was homogeneous. When the echo intensity of the measured nodule was heterogeneous and dominated by a certain echo intensity area, the largest ROI was selected in this echo intensity area (**Figures 4**, **5**). The largest ROI possible was taken when the echo intensity of the measured nodule was heterogeneous without a dominant echo intensity area (**Figure 6**). Calcification, cystic degeneration, and halo were avoided during all of these measurements (**Figure 4**). When measuring the surrounding thyroid tissues, the nonnodular echo areas with the largest proportion of the scanned sections and relatively consistent background echoes were selected, and the ROI that was greater than or equal to that of the nodule was adopted (**Figures 4** **–** **7**). Nodules and thyroid tissues that had the same gain levels at the ROI centers were selected. Abnormal echo areas caused by operators’ technical factors were avoided. All nodules were measured twice, and the final UGSR of the nodule was derived from the means of two measurements, which were calculated as follows:

**Figure 7:**
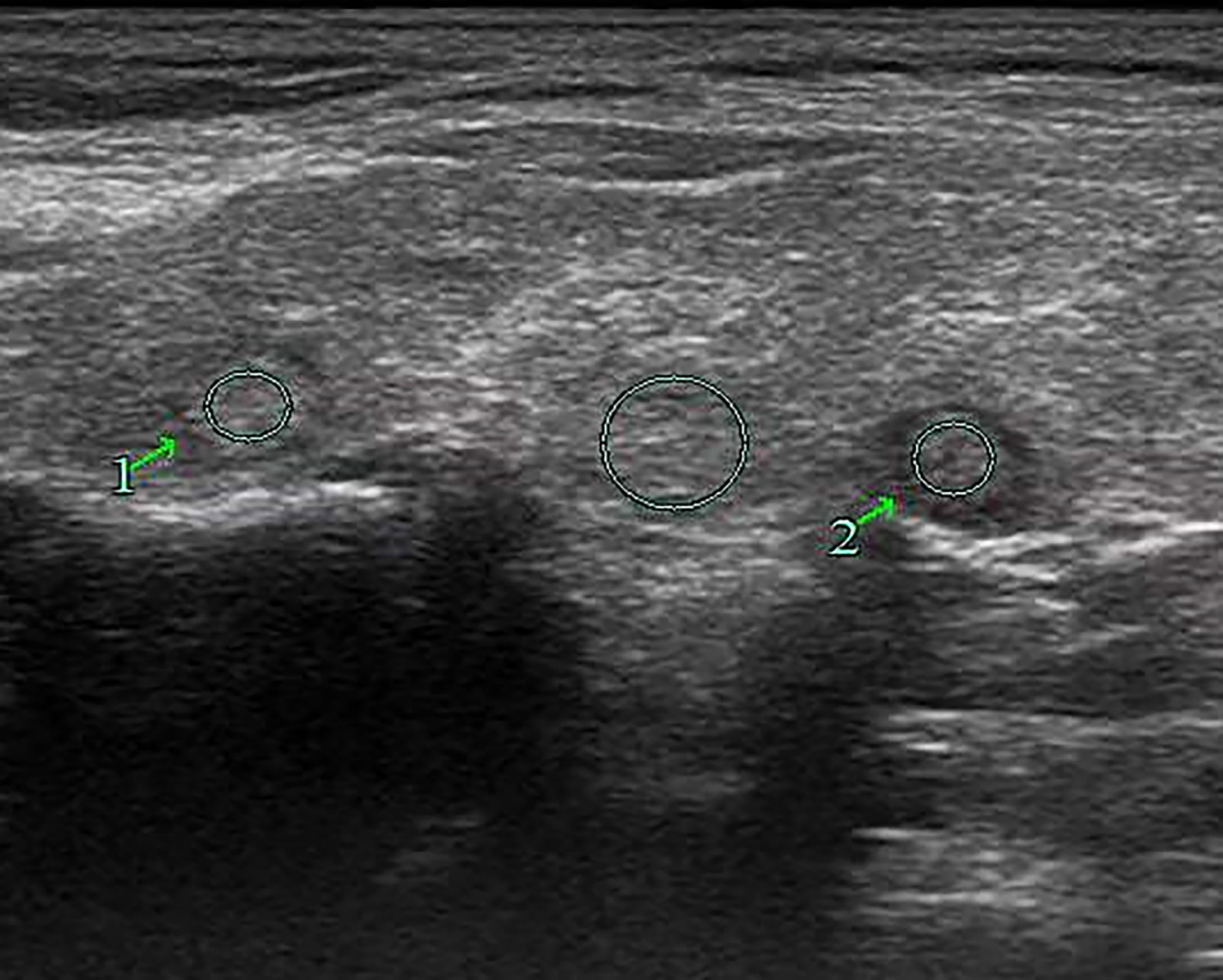
Female patient, 63 years old, 1-week case history of a right-side thyroid nodule, pathology confirmed as nodular goiter with HT. TPO-Ab > 1,300 kU/L, TG-Ab = 46.7 kU/L, UGSR 1 = 100.77/98.43 = 1.024, UGSR 2 = 84.82/98.43 = 0.8617 (medical center A).


$$UGSR_{1}=\frac{nodule\;grayscale\;values\;of\;measurement\;1}{surrounding\;normal\;thyroid\;tissue\;grayscale\;value\;of\;measurement\;1}$$

$$UGSR_{2}=\frac{nodule\;grayscale\;values\;of\;measurement\;2}{surrounding\;normal\;thyroid\;tissue\;grayscale\;value\;of\;measurement\;2}$$

$$UGSR=\frac{UGSR_{1}+UGSR_{2}}{2}$$

### Statistical analysis

Statistical analyses were performed using SPSS 26.0 (IBM Corporation, Armonk, NY, USA) and MedCalc 16.8 (MedCalc, Ostend, Belgium). Data are expressed as mean ± standard deviation or median with interquartile range (IR) depending on whether the data fit the normal distribution. Categorical data are reported as numbers. Continuous variables were analyzed using an independent samples *t*-test or a Mann–Whitney *U* test, while categorical variables were compared using a Pearson’s Chi-squared test or Fisher’s exact test. Receiver operating characteristic (ROC) curves were used to analyze the diagnostic efficiency of UGSR in differentiating between PTMCs and BMNs in patients with HT in medical centers A and B. Differences were considered significant at *p* ≤ 0.05.

## Results

### Distribution of gender, age, size of nodules, TPO-Ab, and TG-Ab levels in PTMCs and BMNs in patients with HT in both medical centers

In both medical centers A and B, the proportion of female patients was significantly higher than that of male patients. The age of the PTMC patients was younger than that of the BMN patients, and the size of the PTMCs was smaller than that of the BMNs. There were no significant differences between the PTMC and BMN patients’ TPO-Ab and TG-Ab levels (**Table 1**).

**Table 1:** Distribution of gender, age, size of nodules, TPO-Ab, and TG-Ab levels in PTMCs and BMNs in patients with HT in both medical centers.

Characteristics	Medical center A		Z/*χ²*	*p*	Medical center B		*Z*/*χ²*	*p*
	PTMCs	BMNs			PTMCs	BMNs		
Gender (*N*)								
Female	342	166	0.368	0.544	298	210	2.251	0.134
Male	27	16			26	19		
Age (years)								
Range	21**~**76	26**~**72			13**~**78	23**~**72		
Median (IR)	45 (36.5, 53.5)	53 (44.5, 58.0)	−5.963	<0.001	44 (36.0, 52.0)	50 (43.0, 56.0)	−5.839	<0.001
Size of nodule (mm)								
Range	4~10	4~10			4~10	4~10		
Median (IR)	6 (5, 7)	7 (5, 9)	−4.606	<0.001	6 (5, 8)	7 (6, 8)	−2.944	0.003
TPO-Ab (kU/L)								
Range	28~1300	28~1,300			28~1,300	28~1,300		
Median (IR)	165.5 (42.3, 1,300.0)	134.4 (28.0, 1,300.0)	−1.384	0.166	186.8 (43.5, 1,300.0)	137.0 (28.0, 654.0)	−1.92	0.055
TG-Ab (kU/L)								
Range	15~500	15~500			15~500	15~500		
Median (IR)	112.8 (47.7, 244.6)	132.8 (55.3, 245.6)	−1.074	0.283	156.1 (49.1, 334.4)	143 (45.2, 341.3)	−0.594	0.552

PTMCs, papillary thyroid microcarcinomas; BMNs, benign micronodules; IR, interquartile range.

### Distribution of UGSR for PTMC and BMN in patients with HT in both medical centers

In both medical centers, the UGSR of PTMC in patients with HT was significantly lower than that of BMN. There were no significant differences in the UGSR of PTMCs in the two medical centers, while there were significant differences in the UGSR of BMNs in the two medical centers. The UGSR of medical center A was lower than that of medical center B (**Table 2**).

**Table 2:** Distribution of UGSR in both medical centers.

UGSR	Medical center A	Medical center B	Z	*p*
PTMCs	0.513 (0.442, 0.592)	0.514 (0.431, 0.625)	−0.815	0.415
BMNs	0.857 (0.677, 0.977)	0.917 (0.705, 1.131)	−3.637	<0.001
Z	−15.564	−17.998		
*p*	<0.001	<0.001		

UGSR, ultrasound grayscale ratio; PTMCs, papillary thyroid microcarcinomas; BMNs, benign micronodules.

The ultrasound images of PTMCs are shown in **Figures 2**, **6**, and the ultrasound images of BMNs are shown in **Figures 3** **–** **5**, **7**.

### Diagnostic efficacy of UGSR in differentiating between PTMCs and BMNs in patients with HT

**Figure 8:**
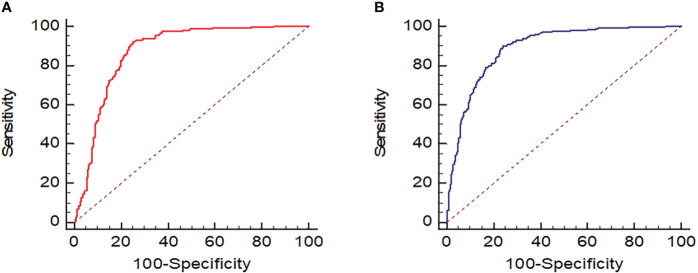
ROC of UGSR in differentiating between PTMCs and BMNs in patients with HT. **(A)** ROC of medical center A; **(B)** ROC of medical center B.

The AUC (**Figure 8**), optimal UGSR threshold, sensitivity, specificity, positive predictive value, negative predictive value, and diagnostic accuracy of UGSR in differentiating between PTMCs and BMNs in patients with HT were 0.870 and 0.889, 0.68 and 0.70, 0.921 and 0.898, 0.747 and 0.759, 0.874 and 0.829, 0.832 and 0.848, and 0.861 and 0.836 for medical centers A and B, respectively (**Table 3**).

**Table 3:** The AUC, the optimal UGSR threshold, and diagnostic efficiency in both medical centers.

Center	AUC	Optimal threshold	Sensitivity	Specificity	PPV	NPV	Accuracy
Center A	0.870	0.68	0.921	0.747	0.874	0.832	0.861
Center B	0.889	0.70	0.898	0.759	0.829	0.8348	0.836

AUC, area under the curve; PPV, positive predictive value; NPV, negative predictive value.

## Discussion

Ultrasound signs such as hypoechoic regions, irregular shapes, microcalcification, and an aspect ratio greater than 1 are important criteria for diagnosing malignant thyroid nodules (5–10). However, these signs are often judged subjectively by observers, especially in the hypoechoic regions (17). Regarding interobserver variability in thyroid nodule echo intensity, Park et al. (18) had a kappa value of 0.57 when analyzing the differences between three radiologists (with 7 to 10 years of training in thyroid imaging). Persichetti et al. (19) used a kappa value of 0.47 when analyzing the differences between seven thyroid imaging specialists (two radiologists and five endocrinologists with more than 15 years of thyroid imaging training). Itani et al. (17) had a kappa value of 0.141–0.355 when analyzing the differences between four radiologists (none with American College of Radiology TI-RADS training, one radiologist with less than 5 years of experience, and three radiologists with more than 15 years of experience). Kim et al. (20) used a kappa value of 0.57 when analyzing five professionals (with 2–8 years of thyroid imaging training) and a kappa value of 0.23 when analyzing four residents (2 second-year residents with 2 months of thyroid imaging training, and 2 fourth-year residents with 6 months of thyroid imaging training). Additionally, a kappa value of 0.12 was used by Kim et al. for the analysis of two second-year residents. It can be seen that there are large differences in the classification of the echo intensity of thyroid nodules by different observers, especially by the junior doctors who have less experience in imaging diagnosis. Quantifying the echo intensity can greatly reduce observers’ subjectivity.

In a quantification study of ultrasound echo intensity of thyroid nodules in 2015, Grani et al. (11) first proposed using UGSR as a method for quantifying the echo intensity of thyroid nodules. However, due to many limitations of their study, including the small sample of malignant nodules, the lack of pathological subtype classification, nodule ungrouping by size, and all nodules being derived from fine needle aspiration cytology rather than pathology, the sensitivity and specificity rates for diagnosing malignant nodules were only 56.7% and 72.0%, respectively. In 2016, Nam et al. (10) proposed using five parameters of the grayscale histogram, including means, skewness, kurtosis, standard deviation, and entropy, as a method for quantifying the echo intensity of thyroid nodules, and this approach’s sensitivity and specificity rates in diagnosing malignant nodules were 56.2%–74.4% and 50.0%–61.8%, respectively, which were values that were far from that of the subjective classification by radiologists, which is 94.5% and 77.6%, respectively. The study of Nam et al. was innovative in that the nodule echo intensity was directly quantified by the five parameters of the histogram. However, these parameters were closely related to factors such as ultrasound model, operator, gain, and dynamics, and any changes in those factors would have resulted in a different parameter value. So, the method by Nam et al. has limitations in the quantification of the value of UGSR. In 2018 (13) and 2021 (14), we conducted a controlled study on using UGSR for PTMC and micronodular goiter (MNG) in a single medical center as well as in two medical centers. The AUC, optimal UGSR threshold, sensitivity, and specificity rates were highly consistent between the two studies, with values of 0.895–0.918, 0.691–0.721, 86.8%–88.1%, and 80.4%–83.3%, respectively. These two studies indicate that UGSR has high diagnostic efficiency and good reproducibility. In 2019, Chen et al. (12) used UGSR to differentiate between PTMC and MNG, and the results had an AUC and optimal UGSR threshold that were highly consistent with our previous studies (13, 14), with values of 0.919 and 0.692, respectively. The comparison of the use of UGSR on the same patient during different examination periods can also support UGSR’s high diagnostic efficiency and reproducibility. We pair analyzed two sets of ultrasonic images from outpatient examinations and preoperative positioning of the same group of patients with PTMC and MNG in 2022. The results show that the AUC and optimal UGSR thresholds of the two sets were highly consistent at 0.860 and 0.856 as well as 0.649 and 0.646, respectively. It was slightly lower than that of our previous studies, which is due to the difference in the participants.

Grani et al. (11), Chen et al. (12) and our previous studies (13, 14) all show that the diagnostic efficiency of UGSR in differentiating between PTMC and MNG was significantly higher than that of a traditional echo intensity grading method (19–22), the latter of which had a sensitivity value of 62%–93.8% and a specificity value of 21.8%–49.7%. These studies were all based on normal thyroid tissues, while this study pair analyzed the UGSR of PTMCs and BMNs in patients with HT in two medical centers, and the results showed that the AUC, optimal UGSR threshold, sensitivity, and specificity values between the two medical centers were highly consistent, and the AUC and optimal UGSR threshold were almost the same as the previous studies. The results indicate that UGSR can still effectively differentiate between PTMCs and BMNs in patients with HT, and the diagnostic efficiency is also relatively stable (13, 14). Compared to previous studies, this study has a lower specificity of 0.747–0.759 but a higher sensitivity of 0.898–0.921. This suggests that UGSR can identify more PTMCs, thereby lowering the rate of missed diagnoses. However, BMNs can be misdiagnosed as PTMCs more often, especially in terms of hypoechoic hyperplastic nodules and subacute thyroiditis in patients with HT. Therefore, when differentiating between PTMCs and BMNs in patients with HT, in addition to UGSR, a comprehensive analysis of other ultrasonic imaging features, such as morphology, aspect ratio, microcalcification, elastography, and the like, is required. Unexpectedly, in this study, there were differences in the UGSR of BMNs between the two medical centers, which was related to the selected samples. We will further expand the medical centers to conduct a controlled study.

There were certain limitations to this study. Firstly, there were some uncertainties in the selection and measurement of ROI in cases with a heterogeneous echo of thyroid tissue due to ultrasound scanning technology or HT factors. Experienced radiologists in both medical centers conducting ROI measurements and adopting the means of two measurements can reduce the deviation as much as possible. Secondly, in this study, nodular goiter accounts for the majority of BMNs, while nodular HT, adenoma, and subacute thyroiditis account for a smaller proportion. However, this is in line with the objective distribution of BMNs. Thirdly, this study did not further analyze the diagnostic efficiency according to different ultrasound models since the models used for thyroid examination in the two medical centers were quite different. Fourthly, this study’s flaw was the lack of QA in checking the consistency/deviation of all ultrasound systems in both hospitals. At present, an ultrasound phantom imaged by all these systems cannot be realized, which is one of the significances of this study, that is, to explore and popularize the value of UGSR, so that the grayscale value can be directly measured on an ultrasound phantom imaged by all these systems in the near future. Furthermore, though some models are the same, the sample sizes are quite different, which does not allow for an accurate analysis. Fifthly, this study is a retrospective analysis, and there may be some selection bias. A prospective, multicenter controlled study would better verify the value and stability of UGSR.

In conclusion, UGSR still has high sensitivity and diagnostic accuracy in differentiating between PTMCs and BMNs in patients with HT, and the diagnostic efficiency of the two medical centers is highly consistent, which provides an important reference for improving TI-RADS. However, due to the low specificity, other ultrasound signs must be considered when analyzing thyroid nodules.

## Data availability statement

The raw data supporting the conclusions of this article will be made available by the authors, without undue reservation.

## Author contributions

NF: writing of original draft, acquisition of data, analysis of data, and substantive translation. PW: writing of original draft, acquisition of data, and analysis of data. XK: acquisition of data and preparation, creation, and presentation of the published work. JX: image processing and analysis of data. JY: writing, reviewing, and editing. FC: revising the manuscript. DO: revising the manuscript. LW: data curation, resources, and visualization. DX: providing oversight, leadership, and mentorship external to the core team. ZH: conception and design of the study, critically reviewing the manuscript for important intellectual content, management, and coordinating the execution of the research activity. All authors listed have made a substantial, direct, and intellectual contribution to the work and approved it for publication.

## Funding

The study was supported in part by the National Natural Science Foundation of China (82071946), the Zhejiang Provincial Natural Science Foundation of China (LSD19H180001, LY20H180001, and LZY21F030001), and the Zhejiang Provincial Medical and Health Technology Project (2022KY110, 2021RC024, and 2020RC091).

## Conflict of interest

The authors declare that the research was conducted in the absence of any commercial or financial relationships that could be construed as a potential conflict of interest.

## Publisher’s note

All claims expressed in this article are solely those of the authors and do not necessarily represent those of their affiliated organizations, or those of the publisher, the editors and the reviewers. Any product that may be evaluated in this article, or claim that may be made by its manufacturer, is not guaranteed or endorsed by the publisher.
